# Diet inclusion of housefly larvae and frass supplemented by Rayabold (enzyme and probiotic) on performance of laying hens and egg quality

**DOI:** 10.1016/j.psj.2025.105544

**Published:** 2025-07-05

**Authors:** Abolfazl Salehizadeh, Mehran Torki, Maryam Darbemamieh, Seyed Davood Sharifi

**Affiliations:** a Animal Science Department, College of Agriculture and Natural Resources, Razi University, Kermanshah, Postal Code: 6715685423, Iran; b Department of Plant Medicine, College of Agriculture and Natural Resources, Razi University, Kermanshah, Iran; c Department of Animal and Poultry Science, Faculty of Agricultural Technology, College of Agriculture and Natural Resources, University of Tehran, Pakdasht, Iran

**Keywords:** Enzyme, Frass, Housefly larvae, Laying hen, Productive performance

## Abstract

This study investigated the effects of dietary housefly larvae and frass (HFLF), with or without a multi-component supplement (Rayabold), on the performance, egg quality, and blood biochemical parameters of Lohmann LSL-Lite laying hens. A total of 192 hens, aged 56 weeks, were assigned to a 10-week feeding trial using a 4 × 2 factorial design with four HFLF inclusion levels (0 %, 5 %, 10 %, and 15 %) and two Rayabold levels (0 % and 0.05 %). A significant interaction was observed for feed intake (FI) (*P* = 0.0001), with hens fed 10 % and 15 % HFLF consuming less feed than those on the control or 5 % diets. Rayabold further reduced FI at higher HFLF levels. Egg production (EP) improved significantly with 10 % and 15 % HFLF (*P* = 0.024), while egg weight (EW) peaked at 5 % HFLF (*P* = 0.003) and was further enhanced by Rayabold (*P* = 0.008). Both HFLF and Rayabold improved feed conversion ratio (FCR), protein efficiency ratio (PER), and energy efficiency ratio (EER) (*P* = 0.0001), with the 15 % HFLF diet yielding the most favorable values. In terms of egg quality, higher HFLF levels (10 % and 15 %) reduced eggshell thickness (*P* = 0.002), while Rayabold increased it (*P* = 0.0007). Haugh unit values were elevated in the 10 % and 15 % HFLF groups (*P* = 0.043). Regarding blood parameters, 10 % HFLF lowered glucose (*P* = 0.0007), and both 10 % and 15 % HFLF increased cholesterol (*P* = 0.001), with Rayabold further elevating it (*P* = 0.030). Triglycerides were reduced by both 10 % HFLF and Rayabold (*P* = 0.050 and *P* = 0.0001, respectively). HFLF decreased AST (*P* = 0.0001) and increased albumin (*P* = 0.006). Complex interactions were noted for ALT and ALP (*P* = 0.0003 and *P* = 0.0001), with Rayabold mitigating the ALT increase seen at 5 % HFLF, and all treatments significantly lowering ALP compared to control. These findings support HFLF, particularly at 10–15 %, as a promising poultry feed ingredient for improving productivity and modulating physiological responses.

## Introduction

The global demand for animal protein is rising steadily, driven by population growth and increasing incomes, especially in developing countries (FAO, 2018; Michalk et al., 2019). This trend places significant pressure on conventional feed ingredients such as soybean meal and fishmeal, which are becoming increasingly expensive and raising sustainability and environmental concerns (Khusro et al., 2012). As a result, there is a growing need to identify alternative protein sources that are both economically viable and environmentally sustainable.

Insects have emerged as a promising solution to this challenge, offering a nutrient-dense and ecologically sustainable feed ingredient (van Huis et al., 2021). Rich in protein, fat, vitamins, and minerals, insects can be efficiently reared on organic waste with minimal resource inputs, including land, water, and feed (Makkar et al., 2014; Gasco et al., 2019; Sorjonen et al., 2019). Among various insect species, housefly larvae (*Musca domestica*, commonly known as maggots) are of particular interest due to their high nutritional value. They typically contain 40–60 % protein and 9–26 % fat, with a balanced amino acid profile comparable to fishmeal, including vital amino acids like lysine, threonine, and methionine, as well as essential vitamins and minerals (Hwangbo et al., 2009; Makkar et al., 2014). Although several studies have examined insect meal in poultry diets (Makkar et al., 2014; Khan et al., 2018; Liu et al., 2021; Sedgh-Gooya et al., 2021), research specifically focused on housefly larvae in laying hen diets is limited (Agunbiade et al., 2007; Zammit and Park, 2024). While evidence supports its effectiveness in replacing conventional ingredients (Zammit and Park, 2024), optimizing inclusion levels is essential to maintain nutrient digestibility and egg quality, particularly eggshell strength (Agunbiade et al., 2007). In addition to their nutritional value, insects contain chitin—a polysaccharide with potential prebiotic effects (Józefiak et al., 2016; de Carvalho et al., 2019). Although most animals lack endogenous chitinase (Shahidi and Abuzaytoun, 2005; Tabata et al., 2017), microbial fermentation of chitin in the gut may promote microbiome balance and immune modulation (Finke, 2007). Furthermore, insect-derived antimicrobial peptides (AMPs) may contribute antibacterial benefits (Dörper et al., 2021).

Insect production also generates frass—a byproduct consisting of larval waste, exoskeletons, uneaten feed, and microbial biomass (Schmitt and de Vries, 2020). While traditionally used as a biofertilizer, frass is gaining recognition as a potential feed ingredient. Studies have shown that black soldier fly and mealworm frass can improve growth performance, nutrient use, and immunity in broilers, aquaculture species, and ruminants (Yildirim-Aksoy et al., 2020a,b; Ayaz et al., 2023). Frass contains valuable components such as chitin, lauric acid, beneficial microbes, and AMPs (Esteban et al., 2001; Powell and Rowley, 2007), although its high ash content may limit inclusion rates in poultry diets. Maximizing nutrient utilization is essential for sustainable poultry production. Feed additives—such as enzymes, probiotics, and prebiotics—can improve gut health, nutrient absorption, and feed efficiency (Slominski, 2011; Islam and Yang, 2017; Al-Khalaifah, 2018; Haider et al., 2024). Rayabold, a multi-component supplement combining enzymes, probiotics, and prebiotics (Khodaei et al., 2022), may help mitigate anti-nutritional effects and support productive performance. This study aims to evaluate the effects of housefly larvae and frass (HFLF), with and without Rayabold supplementation, on productive performance, egg quality, and blood biochemical parameters in Lohmann LSL-Lite laying hens.

## Materials and methods

### Ethics statement

The animal care protocols implemented during the experiment received approval from the ethics committee at Razi University (IR.RAZI.AEC.1404.014), located in Kermanshah, Iran.

### Experimental design and diets

A 10-week feeding trial was carried out to assess the effects of dietary HFLF, with and without a multi-component supplement (Rayabold), on the performance of Lohmann LSL-Lite laying hens. One hundred and ninety-two 56-week-old hens were housed in cages at a density of six birds per replicate (45 × 45 × 45 cm wire cage). A factorial design of 4 × 2 was utilized, comprising four levels of HFLF inclusion (0, 5, 10, and 15 %) and two levels of Rayabold supplementation (0 and 0.05 %), with four replicates per treatment (Table 1). The trial was conducted under controlled environmental conditions, maintaining a 16:8 h light-dark photoperiod (16L:8D), a temperature range of 18–21°C, and a relative humidity of 30–40 %. Feed was provided daily at approximately 120 g per hen, with ad libitum access to water. The full ingredient and chemical composition of the experimental diets, designed to fullfill the nutrient requirements of Lohmann LSL-Lite hens (Lohmann Breeders, 2020), is presented in Table 2. The HFLF was sourced from Fardad Kian Fartak Company (Iran), with the supplier’s laboratory reporting a proximate composition of 22 % crude protein, 1.8 % crude fat, and 16 % crude fiber for the mixture. Moreover, a proximate analysis of the experimental diet samples was performed according to the AOAC (2005) method and is shown in Table 3. Rayabold (Fardad Kian Fartak Company, Iran), a commercially available multi-component feed additive, was incorporated into the diets at either 0 % or 0.05 % to potentially enhance nutrient utilization from the HFLF. This product contains a blend of probiotics (10^9^ CFU/g of *Lactobacillus plantarum, L. delbrueckii, Enterococcus faecium, L. casei, L. rhamnosus, Bifidobacterium bifidum, Bacillus coagulans, B. subtilis, B. licheniformis, Streptococcus salivarius,* and *L. acidophilus*), prebiotics (autolyzed yeast, mannan oligosaccharide, β-glucan, and inulin), and various enzymes including protease (1,200,000 U/kg), lipase (75,000 U/kg), glucose oxidase (30,000 U/kg), hemicellulase (20,000 U/kg), α-amylase (30,000 U/kg), β-amylase (20,000 U/kg), xylanase (20,000 U/kg), β-glucanase (20,000 U/kg), and phytase (10,000 U/kg).

**Table 1:** Experimental treatments.

Treatments	HFLF^1^ (%)	Rayabold supplement^2^ (%)
1	0	0
2	5	0
3	10	0
4	15	0
5	0	0.05
6	5	0.05
7	10	0.05
8	15	0.05

1 HFLF = Housefly larvae meal + Frass (0, 5, 10, and 15 %).

2 Rayabold is a commercial supplement, which contains a blend of probiotics (*Lactobacillus plantarum, L. delbrueckii, Bifidobacterium bifidum, Enterococcus faecium, Streptococcus salivarius, L. casei, L. rhamnosus, Bacillus coagulans, B. subtilis, B. licheniformis*, and *L. acidophilus*), prebiotics (autolyzed yeast, mannan oligosaccharide, β-glucan, and inulin), and a suite of enzymes (protease, lipase, glucose oxidase, hemicellulase, α-amylase, β-amylase, xylanase, β-glucanase, and phytase).

**Table 2:** Ingredients and composition of experimental diets.

Diets				
Ingredients	HFLF0	HFLF5	HFLF10	HFLF15
Corn	59.2	52.5	49.69	45.91
Soybean meal	27.5	27.36	25.98	24.78
Calcium carbonate	10.8	10.84	10.88	10.91
HFLF^1^	0	5	10	15
Bentonite	0	0.79	0	0
Dicalcium phosphate	1	0.97	0.9	0.84
Multi mix	0.5	0.5	0.5	0.5
Rayabold^2^	0.05	0.05	0.05	0.05
Binder toxin	0.1	0.1	0.1	0.1
DL-methionine	0.15	0.19	0.2	0.21
L-lysine	0	0	0	0
Salt	0.2	0.2	0.2	0.2
Oil	0.5	1.5	1.5	1.5
Calculated composition				
ME (kcal/kg)	2700	2670	2670	2670
Crude protein (%)	17	17	17	17
Crude fiber (%)	3.14	3.56	4.06	4.55
Calcium (%)	4.2	4.2	4.2	4.2
Available phosphorus (%)	0.42	0.42	0.42	0.42
Lysine (%)	0.81	0.81	0.79	0.77
Methionine + Cysteine (%)	0.63	0.66	0.66	0.66
Threonine (%)	0.6	0.59	0.58	0.56
Tryptophan (%)	0.203	0.2	0.19	0.17
Arginine (%)	1.1	1.09	1.06	1.03
Isoleucine (%)	0.69	0.66	0.63	0.59
Valine (%)	0.77	0.74	0.7	0.66

1 HFLF = Housefly larvae meal + Frass (0, 5, 10, and 15 %); ME = Metabolizable energy.

2 All diets were combined with or without Rayabold supplement (a blend of probiotics (*Lactobacillus plantarum, L. delbrueckii, Bifidobacterium bifidum, Enterococcus faecium, Streptococcus salivarius, L. casei, L. rhamnosus, Bacillus coagulans, B. subtilis, B. licheniformis*, and *L. acidophilus*), prebiotics (autolyzed yeast, mannan oligosaccharide, β-glucan, and inulin), and a suite of enzymes (protease, lipase, glucose oxidase, hemicellulase, α-amylase, β-amylase, xylanase, β-glucanase, and phytase).

**Table 3:** Chemical composition of experimental diets.

Treatments HFLF^1^ (%)	Rayabold^2^ (%)	Crude protein (%)	Crude fiber (%)	Phosphorous (%)	Ash (%)	Insoluble ash (%)
0	0	17.29	1.52	0.58	17.61	1.55
5	0	16.67	2.43	0.66	19.62	1.30
10	0	17.77	2.47	0.70	18.99	1.07
15	0	17.69	3.47	0.76	19.94	1.41
0	0.05	16.50	1.36	0.48	15.94	1.82
5	0.05	17.87	1.57	0.65	17.93	1.63
10	0.05	17.93	2.96	0.70	20.17	1.20
15	0.05	17.99	3.59	0.69	20.31	1.21

1 HFLF = Housefly larvae meal + Frass (0, 5, 10, and 15 %).

2 A blend of probiotics (*Lactobacillus plantarum, L. delbrueckii, Bifidobacterium bifidum, Enterococcus faecium, Streptococcus salivarius, L. casei, L. rhamnosus, Bacillus coagulans, B. subtilis, B. licheniformis*, and *L. acidophilus*), prebiotics (autolyzed yeast, mannan oligosaccharide, β-glucan, and inulin), and a suite of enzymes (protease, lipase, glucose oxidase, hemicellulase, α-amylase, β-amylase, xylanase, β-glucanase, and phytase).

### Productive performance

To determine feed intake (**FI**), the amount of feed remaining by the conclusion of each week was deducted from the daily feed provided. Egg production (**EP**) and average egg weight (**EW**) were calculated from daily records of egg number and weight. The feed conversion ratio (**FCR**) was then calculated as the quotient of average FI (g) and EW (g). Mortality data were collected throughout the study period and used for necessary data corrections. Protein efficiency ratio (**PER**) was calculated by dividing EW (g) by protein intake (g), with the latter being the product of total FI and the protein content of the feed. Finally, the energy efficiency ratio (**EER**) was determined by dividing the total weight of eggs produced by the metabolizable energy (**ME**) intake. ME intake was calculated by multiplying total FI by the feed's energy content.

### Egg quality traits

Egg quality assessment occurred after the experiment's conclusion, with daily sampling of one egg per replicate over a three-day period. The analyzed parameters included yolk weight, albumen weight, yolk height, albumen height, shell weight, shell thickness, and Haugh unit. The ratio of shell weight to total EW was used to determine shell percentage. Eggshell thickness was determined by averaging measurements taken with a micrometer at three locations on each egg: the large end, the middle (equator), and the small end (pointed end). This triplicate measurement accounts for potential variations in shell thickness across the egg's surface. Yolk height was precisely measured using a tripod micrometer (0.01 mm accuracy; Mitutoyo, Japan). The Haugh unit method was employed to determine albumen quality (Eisen et al., 1962). This method uses the formula: 100 × log (*H* + 7.6 − 1.7W^0.37^), where H is albumen height (mm) and W is EW (g).

### Blood biochemical parameters

At the end of the experimental period (66 weeks), a single bird from each replicate was selected for blood collection via the brachial vein before feeding. Following centrifugation (1008 × *g* for 15 minutes), serum samples were stored at −20°C until analysis. These samples were then analyzed for a suite of biochemical parameters, including glucose, cholesterol, triglycerides, albumin, aspartate aminotransferase (**AST**), alanine aminotransferase (**ALT**), and alkaline phosphatase (**ALP**), using commercially available kits (Pars Azmun, Tehran, Iran).

### Statistical analysis

Statistical analyses were performed on all collected data. Normality was assessed using the Shapiro-Wilk test. A two-way analysis of variance (**ANOVA**), based on a completely randomized 4 × 2 factorial design, was employed to evaluate the effects of four levels of HFLF and two levels of Rayabold supplementation, as well as their interaction. The statistical model used was: Y_ijk_ = μ + A_i_ + B_j_ + AB_ij_ + e_ijk,._ The model incorporated main effects for HFLF (A_i_) and Rayabold (B_j_), as well as an interaction term (AB_ij_), with the observed value represented by Y_ijk_, the overall mean by μ, and the random error by e_ijk.._ These analyses were performed using the general linear model (**GLM**) procedure in SAS 9.4 software (SAS, 2015). The experimental unit was a cage for productive performance and an individual bird for egg quality and blood parameters. Tukey's multiple comparison test was applied for post-hoc mean comparisons, with statistical significance set at *P* ≤ 0.05.

## Results

### Productive performance

**Fig 1:**
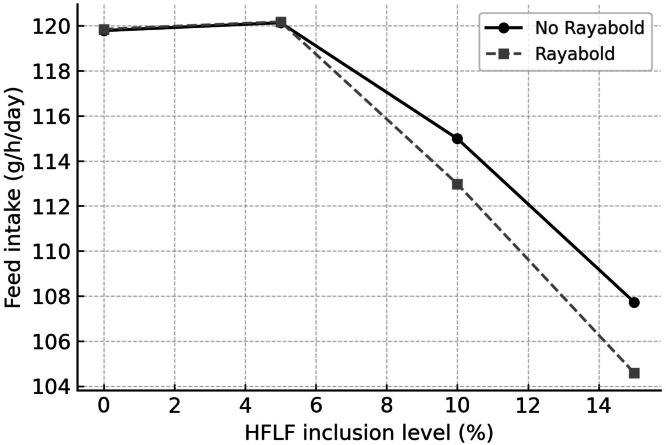
Interaction effect of dietary housefly larvae plus frass (HFLF; 0, 5, 10, and 15 %) and Rayabold supplementation (0 or 0.05 %) on feed intake (g/hen/day) in laying hens.

Table 4 presents the effects of incorporating HFLF with and without Rayabold supplementation on laying hen productive performance. A significant interaction between experimental treatments (HFLF level and Rayabold supplementation) was observed for FI (*P* = 0.0001). Specifically, hens fed diets containing 10 % and 15 % HFLF, with and without Rayabold supplementation, consumed less feed than those on the control and 5 % HFLF diets (Fig 1). At lower HFLF levels (0 % and 5 %), the difference in FI between Rayabold-supplemented and non-supplemented groups was minimal. However, at 10 % and particularly at 15 % HFLF, the Rayabold-supplemented group exhibited a noticeably greater reduction in feed intake compared to the No Rayabold group (Fig 1).

**Table 4:** Influence of dietary housefly larvae plus frass and Rayabold supplement on productive performance of laying hens.

Treatments		FI (g/h/day)	HDEP (%)	EW (%)	FCR (g/g)	PER	EER
HFLF (%)							
0		119.82^a^	89.69^b^	64.97^b^	1.85^a^	3.19^c^	0.20^c^
5		120.17^a^	89.53^b^	67.36^a^	1.79^b^	3.30^bc^	0.21^bc^
10		113.99^b^	95.31^a^	65.37^ab^	1.75^b^	3.37^b^	0.22^b^
15		106.16^c^	95.47^a^	63.35^b^	1.68^c^	3.51^a^	0.23^a^
Rayabold (%)							
0		115.66^a^	93.28	64.25^b^	1.80^a^	3.27^b^	0.21^b^
0.05		114.40^b^	91.72	66.27^a^	1.73^b^	3.41^a^	0.22^a^
Interactions							
HFLF	Rayabold						
0	0	119.79^a^	89.69	63.56	1.89	3.12	0.20
5	0	120.14^a^	91.56	66.19	1.81	3.24	0.21
10	0	114.99^b^	96.88	63.27	1.82	3.24	0.21
15	0	107.73^d^	95.00	64.00	1.68	3.49	0.22
0	0.05	119.85^a^	89.69	66.40	1.81	3.26	0.21
5	0.05	120.18^a^	87.50	68.53	1.76	3.35	0.21
10	0.05	112.98^c^	93.75	67.47	1.68	3.51	0.22
15	0.05	104.59^e^	95.94	62.70	1.67	3.53	0.22
SEM		0.658	7.26	2.92	0.078	0.153	0.056
P value							
HFLF		0.0001	0.024	0.003	0.0001	0.0001	0.0001
Rayabold		0.0001	0.393	0.008	0.0001	0.0001	0.003
HFLF*Rayabold	0.0001	0.726	0.062	0.166	0.158	0.378	

HFLF = Housefly larvae + Frass; FI = Feed intake; HDEP = Hen-day egg production; EW = Egg weight; FCR = Feed conversion ratio; PER = Protein efficiency ratio; EER = Energy efficiency ratio.

Rayabold = Supplement containing prebiotic, probiotic and multi-enzyme.

SEM = Standard error of mean.

Means with different superscripts in columns differ significantly (*P* ≤ 0.05).

Supplementation with HFLF impacted EP in laying hens (*P* = 0.024). Specifically, hens receiving diets containing 10 % and 15 % HFLF demonstrated increased EP compared to those receiving the control diet and the 5 % HFLF diet. Both HFLF and Rayabold supplementation influenced EW. Hens fed the 5 % HFLF diet laid larger eggs than those on the control or 15 % HFLF diets (*P* = 0.003). Additionally, the addition of Rayabold to the diet resulted in an increase in EW in relation to diets that did not contain Rayabold (*P* = 0.008).

The main effects of both HFLF and Rayabold supplementation on FCR, PER, and EER were significant. The inclusion of HFLF significantly decreased FCR in relation to the control diet (*P* = 0.0001). Similarly, diets with Rayabold resulted in lower FCRs than those without (*P* = 0.0001). The 15 % HFLF diet yielded the highest PER and EER values, followed by the 10 % HFLF diet; both were higher than the control (*P* = 0.0001). Rayabold supplementation also increased PER and EER (*P* = 0.0001).

### Egg quality traits

Table 5 details the egg quality traits resulting from the inclusion of HFLF, with and without Rayabold supplementation. The main effects of HFLF and Rayabold supplementation on EW and shell thickness showed differences among treatments. Regarding EW, the diet containing 5 % HFLF resulted in the highest EW, showing a difference compared to the 15 % HFLF treatment, which yielded the lowest EW (*P* = 0.047). Moreover, Rayabold supplementation enhanced EW (*P* = 0.044). The inclusion of 10 % and 15 % HFLF led to a reduction in eggshell thickness compared to both the control and the 5 % HFLF treatments (*P* = 0.002). Rayabold supplementation resulted in an increase in eggshell thickness compared to the control treatment (*P* = 0.0007).

**Table 5:** Influence of dietary housefly larvae plus frass and Rayabold supplement on egg quality traits of laying hens.

Treatments		EW (g)	Yolk W (g)	Albumen W (g)	Shell W (g)	Shell T (10^-2^ mm)	Yolk H (mm)	Albumen H (mm)	HU
HFLF (%)									
0		65.44^ab^	18.10	37.18	10.16	0.38^a^	15.98	3.70	47.83^ab^
5		67.84^a^	18.87	39.57	9.40	0.36^a^	15.34	3.33	42.23^b^
10		65.84^ab^	18.44	37.76	9.64	0.34^b^	15.39	4.44	58.35^a^
15		63.81^b^	17.90	36.14	9.77	0.33^b^	15.57	4.22	57.69^a^
Rayabold (%)									
0		64.72^b^	18.08	36.94	9.70	0.34^b^	15.35	4.04	53.81
0.05		66.75^a^	18.58	38.39	9.78	0.37^a^	15.79	3.81	49.24
Interactions									
HFLF	Rayabold								
0	0	64.02	17.64	36.29	10.09	0.35	15.13	3.82	49.48
5	0	66.67	18.43	38.91	9.33	0.36	15.39	3.68	48.69
10	0	63.72	17.76	36.35	9.62	0.32	15.62	4.46	59.55
15	0	64.46	18.49	36.20	9.77	0.33	15.26	4.21	57.52
0	0.05	66.88	18.56	38.08	10.24	0.40	16.84	3.58	46.19
5	0.05	69.02	19.32	40.22	9.48	0.37	15.30	2.98	35.77
10	0.05	67.96	19.13	39.19	9.65	0.36	15.17	4.43	57.15
15	0.05	63.15	17.31	36.07	9.77	0.35	15.88	4.24	57.85
SEM		3.39	1.33	3.05	0.888	0.026	0.991	1.17	15.79
P value									
HFLF		0.047	0.307	0.061	0.220	0.002	0.389	0.100	0.043
Rayabold		0.044	0.197	0.107	0.761	0.0007	0.127	0.489	0.322
HFLF*Rayabold	0.238	0.100	0.691	0.995	0.186	0.067	0.870	0.753	

HFLF = Housefly larvae + Frass; EW = Egg weight; *W* = Weight; *H* = Height; HU = Haugh unit.

Rayabold = Supplement containing prebiotic, probiotic and multi-enzyme.

SEM = Standard error of mean.

Means with different superscripts in columns differ significantly (*P* ≤ 0.05).

The main effect of HFLF on Haugh units was also observed (*P* = 0.043). Specifically, treatments containing 10 % and 15 % HFLF demonstrated higher Haugh unit values than the 5 % HFLF treatment. Yolk weight, albumen weight, shell weight, yolk height, and albumen height showed no difference between treatments (*P* > 0.05).

### Blood parameters

The effects of incorporating HFLF, with and without Rayabold supplementation on blood parameters are summarized in Table 6. The main effect of HFLF on blood glucose concentration was observed (*P* = 0.0007). Hens that were fed a diet containing 10 % HFLF showed lower glucose concentrations than those on the other diets.

**Table 6:** Influence of dietary housefly larvae plus frass and Rayabold supplement on blood parameters of laying hens.

Treatments		Glucose (mg/dL)	Cholesterol (mg/dL)	Triglyceride (mg/dL)	AST (U/L)	ALT (U/L)	ALP (U/L)	Albumin (g/dL)
HFLF (%)								
0		166.13^a^	130.75^b^	1126.50^ab^	183.00^a^	24.37	705.25^ab^	2.26^b^
5		157.13^a^	129.88^b^	1118.00^bc^	157.13^c^	21.37	820.00^a^	2.45^a^
10		143.38^b^	162.88^a^	1103.25^c^	150.38^c^	21.50	651.00^b^	2.39^a^
15		160.38^a^	160.50^a^	1137.63^a^	173.38^b^	20.62	725.00^ab^	2.42^a^
Rayabold (%)								
0		154.31	137.94^b^	1151.13^a^	157.88^b^	23.31^a^	808.75^a^	2.39
0.05		159.19	154.06^a^	1091.56^b^	174.06^a^	20.62^b^	641.88^b^	2.37
Interactions								
HFLF	Rayabold							
0	0	163.75	120.25	1151.25	179.75	21.75^b^	988.75^a^	2.30
5	0	154.00	118.50	1151.75	152.00	27.00^a^	798.25^b^	2.45
10	0	137.25	153.50	1125.75	138.75	23.75^ab^	704.00^bc^	2.33
15	0	162.25	159.50	1175.75	161.00	20.75^bc^	744.00^bc^	2.50
0	0.05	168.50	141.25	1101.75	186.25	27.00^a^	421.75^d^	2.23
5	0.05	160.25	141.25	1084.25	162.25	15.75^c^	831.75^b^	2.45
10	0.05	149.50	172.25	1080.75	162.00	19.25^bc^	598.00^c^	2.45
15	0.05	158.50	161.50	1099.50	185.75	20.50^bc^	706.00^bc^	2.35
SEM		9.69	19.83	17.61	8.96	3.27	112.64	0.103
P value								
HFLF		0.0007	0.001	0.005	0.0001	0.135	0.044	0.006
Rayabold		0.167	0.030	0.0001	0.0001	0.029	0.0003	0.500
HFLF*Rayabold		0.442	0.709	0.265	0.127	0.0003	0.0001	0.076

HFLF = Housefly larvae + Frass; AST = Aspartate aminotransferase; ALT = Alanine aminotransferase; ALP = Alkaline phosphatase (ALP).

Rayabold = Supplement containing prebiotic, probiotic and multi-enzyme.

SEM = Standard error of mean.

Means with different superscripts in columns differ significantly (*P* ≤ 0.05).

Differences were noted in blood cholesterol and triglyceride concentrations due to the main effects of HFLF and Rayabold. Diets containing 10 % and 15 % HFLF resulted in elevated blood cholesterol concentrations relative to the control and 5 % HFLF diets (*P* = 0.001). This elevation was further augmented by Rayabold supplementation (*P* = 0.030). Conversely, the 10 % HFLF diet resulted in the lowest triglyceride concentration, lower than the control and 15 % HFLF diets (*P* = 0.005). Rayabold supplementation also reduced blood triglyceride concentration (*P* = 0.0001).

**Fig 2:**
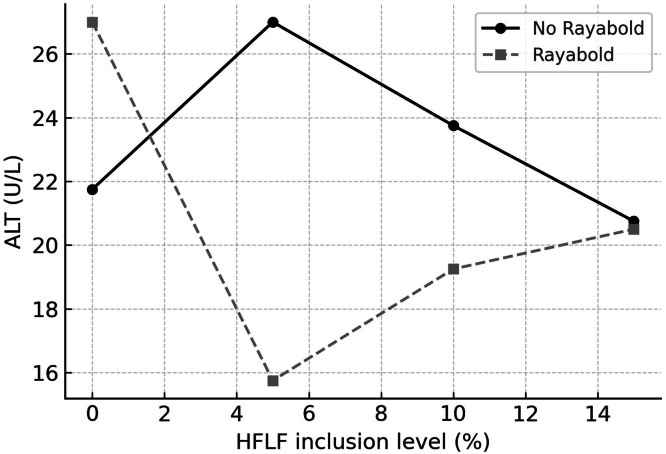
Interaction effect of dietary housefly larvae plus frass (HFLF; 0, 5, 10, and 15 %) and Rayabold supplementation (0 or 0.05 %) on serum alanine aminotransferase (ALT; U/L) activity in laying hens.

**Fig 3:**
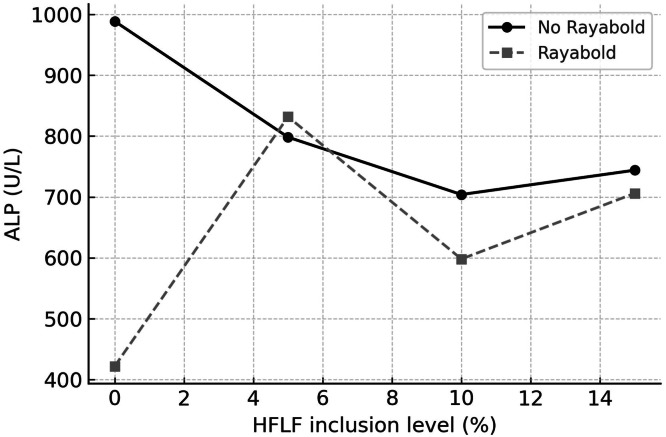
Interaction effect of dietary housefly larvae plus frass (HFLF; 0, 5, 10, and 15 %) and Rayabold supplementation (0 or 0.05 %) on serum alkaline phosphatase (ALP; U/L) activity in laying hens.

The primary effects of HFLF and Rayabold on blood AST concentration were observed. Hens that consumed diets with 5 %, 10 %, and 15 % HFLF showed reduced AST content in comparison to the control group (*P* = 0.0001). Rayabold supplementation, however, increased AST concentration (*P* = 0.0001). Significant interaction effects were observed between the experimental treatments on blood ALT (*P* = 0.0003) and ALP (*P* = 0.0001) contents. Specifically, while both the 5 % HFLF treatment alone and Rayabold supplementation alone led to an increase in ALT concentration relative to the control group, the combined treatment of 5 % HFLF with Rayabold resulted in a *lower* ALT concentration than the control (Fig 2). Regarding ALP, in the absence of Rayabold, ALP levels commenced at their highest point at 0 % HFLF and generally decreased with increasing HFLF inclusion, reaching a nadir at 10 % HFLF (Fig 3). In contrast, Rayabold supplementation resulted in markedly lower ALP levels at 0 % HFLF compared to the non-supplemented group. Intriguingly, at 5 % HFLF, Rayabold supplementation led to an increase in ALP, bringing its concentration closer to that of the non-supplemented group at this HFLF level (Fig 3). Beyond 5 % HFLF, Rayabold generally contributed to lower ALP levels compared to the non-supplemented counterparts at equivalent HFLF concentrations (Fig 3). The main effect of HFLF on albumin concentration was observed (*P* = 0.006). Diets supplemented with 5 %, 10 %, and 15 % HFLF resulted in increased albumin concentrations compared to the control diet.

## Discussion

### Growth performance

This study investigated the effects of dietary inclusion of HFLF, with and without Rayabold supplementation, on laying hen performance, egg quality, and blood parameters. A key finding was a significant interaction between HFLF inclusion level and Rayabold supplementation on FI. Specifically, higher inclusion levels of HFLF (10 % and 15 %) significantly reduced FI, an effect further exacerbated by Rayabold supplementation.

Several factors may explain the observed FI reduction with increasing HFLF inclusion. Firstly, the high crude fiber content (16 %) of the HFLF mixture likely contributed to increased satiety. Dietary fiber is known to increase digesta viscosity and slow gastrointestinal transit time, thus promoting satiety and reducing appetite (Mateos et al., 2012; Jha and Mishra, 2021; Akhlaghi, 2024). Secondly, palatability of diets containing higher HFLF levels may have been compromised. The darker coloration of insect larvae can influence palatability and visual appeal, potentially leading to feed rejection, especially at higher inclusion levels (Tabeekh, 2015). This is consistent with studies on black soldier fly larvae meal (**BSFLM**), where darker pigmentation and higher inclusion rates resulted in reduced FI (Khosravinia, 2007; Marono et al., 2017; Bovera et al., 2018). Additionally, similar FI reductions have been reported with maggot meal inclusion (Atteh and Adeyoyin, 1993; Okah and Onwujiariri, 2012; Khan et al., 2016, 2018).

However, the literature presents conflicting results regarding insect meal and FI. While some studies reported increased FI with housefly maggot products (Hamani et al., 2022; Zammit and Park, 2024), others found no significant effect (Akpodiete et al., 1998; Agunbiade et al., 2007). Adams and Koutsos (2024) reported no change in FI with BSF frass in broiler diets. Conversely, in catfish, BSF frass inclusion at 20 % and 30 % resulted in higher FI compared to the control (Yildirim-Aksoy et al., 2020a). Notably, no studies specifically investigating HFLF were found. These discrepancies underscore the complex interplay of factors influencing FI, including insect species, processing methods (e.g., fresh, dried, extracted), inclusion levels, and bird genetics.

The further reduction in FI observed with Rayabold supplementation is likely related to enhance nutrient digestibility facilitated by the enzymes present in the supplement. This may lead to increased nutrient absorption and a consequent downregulation of appetite. This aligns with findings by Ravindran (2012), who highlighted the role of exogenous enzymes in improving nutrient utilization and potentially affecting FI. Furthermore, studies on broilers have reported reduced FI with synbiotic supplementation, attributed to improved feed efficiency through modulation of the intestinal microflora, enhanced intestinal integrity, and immune stimulation (Al-Sultan et al., 2016; Nisar et al., 2021).

Despite the reduced FI at higher HFLF inclusion levels, significant improvements were observed in FCR, PER, and EER. This indicates more efficient nutrient utilization from the HFLF diets compared to the control. These findings are consistent with previous reports on the beneficial effects of HFL on FCR in poultry (Okah and Onwujiariri, 2012), although contrasting with Adams and Koutsos (2024), who reported no change in FCR with BSF frass in broilers. The nutrient profile of frass, including lauric acid, chitin, and antimicrobial peptides, may contribute to improved animal health and consequently increased nutrient absorption (Adams and Koutsos, 2024). Moreover, HFL is a rich source of high-quality protein with a complete essential amino acid profile comparable to or exceeding that of soybean meal (Makkar et al., 2014). This, coupled with potentially enhanced digestibility, likely contributes to the higher PER. The high fat content of HFL, primarily readily digestible medium-chain fatty acids (**MCFA**), likely plays a significant role in improved energy utilization, consistent with observations in tilapia fed frass-containing diets (Yildirim-Aksoy et al., 2020b). This contrasts with studies reporting reduced EER and PER in birds fed BSFLM (Nampijja et al., 2023), attributed to decreased FI, suggesting defatting BSFLM could be beneficial. Rayabold's positive effects on FCR, PER, and EER further support its role in enhancing nutrient utilization, compensating for lower FI by maximizing the use of available nutrients (Slominski, 2011).

A key finding was the significant increase in EP with 10 % and 15 % HFLF compared to the control and 5 % HFLF diets, highlighting improved nutrient utilization and the potential of HFLF as a valuable protein and energy source. The specific amino acid profile of HFLF may better meet the hens' requirements for EP. EW was more nuanced; while the 5 % HFLF diet resulted in greater EW than the control and 15 % diets. Rayabold supplement consistently increased EW across all treatments, suggesting a crucial role for Rayabold in supporting EW, possibly through improved nutrient absorption and availability (Ravindran, 2012; Jha and Mishra, 2021). The lower EW at 15 % inclusion, despite increased EP, could be related to the reduced FI at this level. The 5 % inclusion level may represent an optimal balance between EP and EW without significantly reducing FI. These findings are partially consistent with studies observing increased EW and EP with housefly maggot extract (Zammit and Park, 2024), and align with other studies showing positive effects of insect meal on EP parameters (Parshikova et al., 1981) or at least no negative impact at certain inclusion levels (Agunbiade et al., 2007).

### Egg quality traits

While no statistically significant effects were observed on albumen weight, albumen height, yolk weight, yolk height, and eggshell weight across the experimental treatments, a notable finding was the reduction in eggshell thickness with increasing levels of HFLF inclusion. This observation is consistent with some previous research indicating detrimental effects of elevated HFL meal inclusion rates on shell quality (Agunbiade et al., 2007). Conversely, other studies have reported no significant impact of insect meal on shell quality (Sedgh-gooya et al., 2021), while some recent investigations have demonstrated improved eggshell quality with housefly maggot extract (Zammit and Park, 2024), and BSFLM at specific inclusion levels (Liu et al., 2021). This discrepancy underscores the complex interplay between insect meal composition, inclusion level, and other dietary factors.

A potential mechanism for the observed reduction in shell thickness relates to mineral imbalances, specifically a lower calcium content in HFL compared to traditional protein sources such as fishmeal (Agunbiade et al., 2007). Critically, both HFL and its associated frass contribute to the overall dietary phosphorus content (Basri et al., 2022). This is a salient point, as the combined phosphorus from HFL and frass can exacerbate the potential for mineral imbalances. As emphasized by Keshavarz (2003), maintaining an appropriate calcium:phosphorus ratio is crucial for optimal shell formation. The increased phosphorus from the combined HFL and frass, without meticulous dietary calcium adjustment, could therefore disrupt this balance, resulting in thinner shells. However, the significant positive effect of Rayabold supplementation on eggshell thickness suggests that the prebiotics, probiotics, and enzymes contained within this supplement may have mitigated the negative effects of HFL and frass on shell formation. Existing literature demonstrates that probiotics, prebiotics, and particularly synbiotics can effectively enhance eggshell quality by improving calcium absorption and promoting a balanced gut microbiome (Abdelqader et al., 2013). These researchers posit that prebiotics are fermented by gut bacteria, leading to a reduction in intestinal pH and increased production of short-chain fatty acids, thereby enhancing calcium solubility and absorption. Additionally, enzymes can improve the digestibility of dietary components, potentially releasing more calcium for shell formation (Olgun et al., 2018). These combined effects likely contributed to the observed increase in eggshell thickness in the Rayabold-supplemented groups.

Interestingly, despite the negative impact on shell thickness, higher HFLF inclusion levels resulted in increased Haugh unit values. Haugh unit, primarily determined by albumen height, reflects the quality of egg white proteins. This suggests that HFLF may have positively influenced albumen characteristics, potentially due to its rich amino acid profile (Makkar et al., 2014). This finding is partially corroborated by Zammit and Park (2024), who reported improved Haugh unit with housefly maggot extract. However, this contrasts with the findings of Agunbiade et al. (2007), who observed no significant effect of maggot meal on Haugh unit. This discrepancy may be attributable to variations in the type of insect meal used, processing methods, inclusion levels, or other dietary components.

### Blood parameters

Blood biochemical parameters serve as crucial indicators of avian health status (Lumeij, 2008), providing valuable tools for evaluating the safety and physiological effects of novel feed components. In the present study, hens fed a diet incorporating 10 % HFLF exhibited a statistically significant reduction in blood glucose levels compared to all other treatment groups. This observed hypoglycemic effect contrasts with some studies utilizing BSFLM, which reported no significant alterations in glucose concentrations (Marono et al., 2017). However, other research suggests a link between insect-derived chitin, a major component of insect exoskeletons, and carbohydrate metabolism. This aligns with findings suggesting that chitin and its derivative, chitosan, can modulate glucose absorption within the gastrointestinal tract, potentially leading to lower postprandial glucose levels (Tzeng et al., 2022). For instance, Sajjad et al. (2024) reported the lowest glucose concentrations in broiler chickens fed a 12 % BSFLM diet relative to the control group. The observed reduction in blood glucose in the present study may be attributable to the high fiber content (16 %) of the HFLF. Fiber contributes to stable blood sugar levels and overall metabolic health by slowing digestion, enhancing insulin sensitivity, and increasing feelings of fullness (de Leeuw et al., 2004). Similarly, Zhang et al. (2023) observed the lowest plasma glucose content in broilers fed an 8 % crude fiber diet compared to groups receiving diets containing 2, 5, and 11 % fiber. Furthermore, Yildirim-Aksoy et al. (2023) demonstrated that the addition of BSF frass in the diet resulted in significantly lower glucose levels in the treated groups relative the control group. These findings further support the hypothesis that dietary fiber content can significantly influence glucose metabolism.

A key observation in this study was the contrasting influence of HFLF and the Rayabold supplement on lipid profiles. While a 10 % inclusion of HFLF significantly reduced triglyceride concentrations compared to both the control and the 15 % inclusion level, it concurrently elevated cholesterol levels at both 10 % and 15 % inclusion. This dual effect may be attributed to the diverse fatty acid and sterol profiles inherent in insect meals. As highlighted by Yildirim-Aksoy et al. (2023), cholesterol is a predominant sterol in insects, which may explain the observed increase in cholesterol levels. This is consistent with findings by Ekpo et al. (2009), who reported an average cholesterol content of 3.6 % in the lipid fraction of the termite *Macrotermes bellicosus* and the caterpillar *Imbrasia belina*. Similarly, Hu et al. (2017) observed increased plasma cholesterol in yellow catfish (*Pelteobagrus fulvidraco*) fed diets with high BSFLM inclusion. However, contrasting results have also been reported. According to a research the lowest cholesterol levels were observed in broilers fed a 12 % BSFLM meal diet compared to the control, suggesting a potential hypolipidemic effect of certain insect meals (Sajjad et al., 2024). This is further supported by Marono et al. (2017), who demonstrated significantly lower cholesterol and triglyceride levels in hens fed BSFLM compared to those fed soybean meal. These lipid-lowering effects, particularly those associated with HILM, have been linked to the presence of chitin (Hossain and Blair, 2007). These authors demonstrated a dose-dependent reduction in serum cholesterol and triglycerides in broilers fed diets supplemented with commercial chitin (0, 25, 50, and 75 g/kg), with the most consistent reduction at 50 g/kg. The proposed mechanism for chitin's hypolipidemic action involves its positive charge, which attracts negatively charged bile acids and free fatty acids, thereby impeding their absorption (Prajapati and Patel, 2010). This mechanism may contribute to the hypolipidemic effects observed in the present study with HFLF and in other studies employing insect meals. In contrast to HFLF, Rayabold supplementation increased serum cholesterol and decreased serum triglycerides compared to the control. The reduction in triglycerides with Rayabold aligns with the expected action of lipases, which promote lipid hydrolysis and potentially enhance fatty acid absorption and utilization.

Hepatic function, as assessed by AST, ALT, and ALP activities, was influenced by the dietary treatments. Notably, all levels of HFLF inclusion significantly reduced AST concentrations compared to the control group, suggesting a potential hepatoprotective effect. This reduction may be attributed to the presence of bioactive compounds within the HFLF, like chitin, lauric acid and AMPs, which could mitigate oxidative stress and subsequent hepatic damage (Dörper et al., 2021; Adams and Koutsos, 2024). However, the concurrent supplementation of Rayabold counteracted this effect, resulting in a significant increase in AST levels. This interaction indicates that Rayabold may modulate liver enzyme activity in the presence of HFLF. Regarding ALP activity, all HFLF treatments, irrespective of Rayabold inclusion, significantly reduced ALP concentrations compared to the control. This reduction suggests a potential positive influence on bone metabolism or other physiological processes associated with ALP activity. Furthermore, Rayabold mitigated the increase in ALT activity observed with 5 % HFLF, suggesting a potential synergistic effect in modulating hepatic function. Studies have reported varying effects of insect-derived ingredients on liver enzymes. No significant changes in liver enzymes were observed in laying hens and broiler chicks fed mealworm meal, BSFLM, and earthworm meal, respectively (Marono et al., 2017; Biasato et al., 2018; Zang et al., 2018). Conversely, age-related changes in ALP and ALT during the grower phase have been documented, with both enzymes decreasing in response to an 8 % maggot meal inclusion compared to 4 % and the control (Elahi et al., 2020). The lack of influence of dietary frass on serum ALT and ALP activities has also been reported, suggesting that frass may not negatively impact hepatic health (Yildirim-Aksoy et al., 2023). This is supported by similar findings in hybrid tilapia fed up to 30 % dietary frass (Yildirim-Aksoy et al., 2020b). Reduced AST and ALT activity has been observed in laying hens fed low levels of silkworm meal (up to 0.05 %) (Kim et al., 2020), while the partial or complete replacement of soybean meal has been shown to have no effect on liver enzymes (Ullah et al., 2017), a finding corroborated by other studies (Lalev et al., 2023) and the present study.

The inclusion of HFLF led to a statistically significant increase in albumin concentration in the treated groups when contrasted with the control group. Plasma proteins, in general, are essential for maintaining body homeostasis, with albumin serving as a vital source of amino acids for protein synthesis. While some studies have reported no significant effects on blood total protein and albumin concentrations following the inclusion of insect meals in poultry diets, suggesting comparable bioavailability of dietary protein (Ullah et al., 2017; Kim et al., 2020; Lalev et al., 2023), the present findings indicate a different outcome. The observed increase in albumin levels in hens fed HFLF suggests enhanced protein utilization and/or improved hepatic function related to protein synthesis. This elevation in albumin levels associated with HFLF supplementation provides further evidence of the nutritional value of this insect meal as a viable protein source, indicating enhanced protein utilization and synthesis.

## Conclusions

This study demonstrates the potential of HFLF as a valuable protein source in laying hen diets. While higher inclusion levels (10 % and 15 %) of HFLF, particularly in conjunction with Rayabold supplementation, resulted in decreased FI, they also yielded significant improvements in FCR, PER, EER, EP. Although these higher inclusion levels negatively impacted eggshell thickness, potentially due to mineral imbalances, they positively influenced Haugh unit scores, indicating enhanced internal egg quality. Notably, the 10 % HFLF diet demonstrated beneficial effects on metabolic parameters, lowering blood glucose and triglyceride levels, albeit with a concomitant increase in cholesterol. Regarding hepatic enzymes, HFLF generally reduced AST concentrations, while Rayabold supplementation had the opposite effect. Complex interactions were observed for ALT and ALP; Rayabold mitigated the increase in ALT associated with the 5 % HFLF diet, and all treatments significantly reduced ALP concentrations compared to the control. In conclusion, HFLF demonstrates considerable promise as a sustainable and potentially valuable feed ingredient for laying hens. However, further research is warranted to optimize inclusion levels, particularly with regard to mitigating the observed negative effects on eggshell quality and modulating blood lipid profiles to achieve a more balanced metabolic outcome. Additionally, future studies should investigate the effects of HFLF across a broader range of hen ages to fully understand its comprehensive metabolic responses throughout the laying cycle.

## Disclosures

The authors declare that they have no known competing financial interests or personal relationships that could have appeared to influence the work reported in this paper.
